# Comparison of miRNA expressions among benign, premalignant and malignant lesions of the larynx: could they be transformation biomarkers?

**DOI:** 10.1186/s40463-021-00497-y

**Published:** 2021-02-27

**Authors:** Fatma Ruya Tuncturk, Ibrahim Akalin, Lokman Uzun, Tulay Zenginkinet

**Affiliations:** 1 Department of Otolaryngology, Head and Neck Surgery, Istanbul Medeniyet University Faculty of Medicine, Istanbul, Turkey; 2 Dr. Behçet Uz Child Disease and Pediatric Surgery Training and Research Hospital Department of Otolaryngology-Head Neck Surgery, İsmet Kaptan Mh, Sezer Doğan Sok No:11, 35210, Konak/Izmir, Turkey; 3 Department of Medical Genetics, Istanbul Medeniyet University Faculty of Medicine, Istanbul, Turkey; 4 Current Address: Maltepe University Faculty of Medicine Department of Medical Genetics, Istanbul, Turkey; 5 Department of Medical Pathology, Istanbul Medeniyet University Faculty of Medicine, Istanbul, Turkey

**Keywords:** miRNA, Larynx, Carcinoma, Leukoplakia, Hyperplasia, Biomarker

## Abstract

**Background:**

The malignancy potential of the laryngeal lesions are one of the major concerns of the surgeons about choosing the treatment options, forming surgical margins, deciding the follow-up periods. Finding a biomarker to overcome these concerns are ongoing challenges and recently microRNAs (miRNAs) are attributed as possible candidates since they can regulate gene expressions in the human genome. The objective of our study was to investigate their capability as a transformation biomarker for malignant laryngeal lesions.

**Materials and methods:**

We investigated mature miRNA expressions in paraffin-embedded surgical specimens of human laryngeal tissues grouped as benign, premalignant or malignant (*n* = 10 in each). miRNA profiling was carried out by quantitative Real-Time polymerase chain reaction (RT-qPCR) and data were analyzed according to fold regulation.

**Results:**

Our results demonstrated that 9 miRNAs were upregulated as the lesions become more malignant. Among them *Hs_miR-183_5p, Hs_miR-155_5p, and Hs_miR-106b_3p* expressions were significantly 4.16 (*p* = 0.032), 2.72 (*p* = 0.028) and 3.01 (*p* = 0.022) fold upregulated respectively in premalignant lesions compared to the benign lesions. Moreover, their expressions were approximately 2.76 fold higher in the malignant group than in the premalignant group compared to the benign group. Besides them, significant 7.57 (*p* = 0.036), 4.45 (*p* = 0.045) and 5.98 (*p* = 0.023) fold upregulations of *Hs_miR-21_5p, Hs_miR-218_3p, and Hs_miR-210_3p* were noticed in the malignant group but not in the premalignant group when compared to the benign group, respectively.

**Conclusion:**

MiRNAs might have important value to help the clinicians for their concerns about the malignancy potentials of the laryngeal lesions. *Hs_miR-183_5p, Hs_miR-155_5p, and Hs_miR-106b_3p* might be followed as transformation marker, whereas *Hs_miR-21_5p, Hs_miR-218_3p, and Hs_miR-210_3p* might be a biomarker prone to malignancy.

## Background

Laryngeal carcinoma is one of the most common malignancies in the head and neck region with a good prognosis when in the early stages [1]. However, in advanced stages, responses are weak despite the diverse and novel treatment modalities [2]. The 5-year survival rate of patients with stage 1 laryngeal cancer can reach up to 90%, whereas the 5-year survival rate of the patients with stage 4 laryngeal carcinoma does not even reach to 10% [2]. Traditionally, laryngeal carcinomas are staged according to ‘TNM classification’ and a ‘histopathological grading system’. But, even they are same tumor type within the same tumor classification and at the same localization, the laryngeal tumors that are treated with the same modalities could represent different prognoses. Therefore, the malignancy potential of any laryngeal lesion is important to know to designate the medical or surgical treatment options. Nonetheless, underlying immunohistochemical, pathophysiological or genetic factors such as post-transcriptional regulators could interfere with a malignant transformation that is needed to be investigated.

MicroRNAs (miRNA) are endogenous, small, non-coding RNAs that are 21–24 nucleotides in length and known to regulate gene expression by silencing target transcripts via complement base-pairing in various pathways including embryogenesis, development, differentiation, and apoptosis [3]. miRNAs are also reported to regulate other pathological and physiological processes including cancer [4–6]. Since the protooncogenic and tumor-suppressive effects of miRNAs have been shown, miRNAs have also been investigated in head and neck cancers beside others [6]. Hu et al. have shown a significant correlation between miRNA expression and the factors affecting cancer stages and grades such as lymph node metastasis and distant site metastasis [7]. The relationships of miRNAs expressions with survival rates and prognosis have also been reported in terms of cigarette and/or alcohol consumption besides the demographic features. Though, recent studies have compared premalignant and malignant lesions in head and neck cancers, particularly in oral cavity tumors such as Yu et al. has stated miR-21, miR-106b and miR-375 as novel potential biomarkers [7–9]. Moreover, when compared to healthy controls Ayaz et al*,* has reported significant upregulation and downregulation of 17 (including miR-21) and 9 miRNAs in laryngeal squamous cell carcinoma, particularly [8]. The given studies held healthy subjects or specimens as the control group and reported significant results when compared to healthy controls. Those previously known miRNAs expressed in malignant laryngeal lesions was our rationale to investigate them within our study, since we aimed to elucidate whether there is/are particular miRNA(s) among them could be more prone to be a biomarker of transformation across the benign, premalignant, malignant laryngeal tumors when compared to the benign laryngeal lesions.

## Materials and methods

Here, *Hs_miR-21_5p, Hs_miR-106b_3p, Hs_miR-375_5p, Hs_miR-155_5p, Hs_let7a_5p, Hs_miR-210_3p, Hs_miR-425_3p, Hs_miR-183_5p,* and *Hs_miR-218_3p* expressions were investigated within paraffin-embedded specimens that were obtained from patients who underwent surgery because of laryngeal lesions between the years 2012 and 2015. After local ethical committee approval (2014/0196), informed consent was obtained from the patients. The samples were analyzed within 3 groups as benign (vocal cord polyps; *n* = 10), premalignant (moderate-high-grade dysplasia; *n* = 10) and malignant (squamous cell carcinoma; *n* = 10). Patients who had previous cancer history or had been treated with radiochemotherapy were all excluded. One expert pathologist reviewed 5-μm sections containing the lesions of interest from the FFPE samples. The sections were transferred to the genetics laboratory in suitable conditions.

### Isolation of miRNA-enriched total RNA and complementary DNA synthesis

miRNAs were isolated from the paraffin-embedded tissues of the patients using the miRNeasy FFPE Kit (Qiagen) according to the manufacturer’s protocol. Briefly, xylene and ethanol (%96–100) were used to remove paraffin before total RNA isolation. Next, the pellet was treated with 10 μl of proteinase K. The fully deparaffinized and lysed laryngeal tumor supernatant was transferred to a new microcentrifuge tube and DNase I was added to eliminate the DNA content. Then, RNeasy MinElute Spin Column (Qiagen) and relevant buffers of the kit were used with subsequent centrifugation and flow-through steps at 8000 g for 15 s. Finally, miRNA-enriched total RNA was eluted with 14 μl of RNase-free water.

***c***DNAs were randomly primed from 5 μg of miRNA-enriched total RNA with miScript II Reverse Transcription (Rt) Kit (Qiagen). Briefly, reverse transcription PCR was performed with 4 μl of 5x miScript HiSpec Buffer, 2 μl of 10x Nucleic Acid Mix, 1 μl of miScript Reverse Transcriptase Mix, 8 μl of RNase-free water and 5 μl of template RNA, with a total of volume of 20 μl. The Rt reaction was incubated at 37 °C for 60 min and 95 °C for 5 min. The cDNA was then diluted with 200 μl of nuclease-free water for further use in real-time PCRs.

### Real-time PCR

Nine miRNAs covering a variety of miRNA sequences were selected, and mature miRNA expression was determined via quantitative real-time polymerase chain reaction (RT-qPCR) with a QuantiTech SYBR Green PCR Kit (Qiagen) on the Rotor-Gene® Q (Qiagen, USA) instrument using the 2.1.0.9 software. RT-PCR was performed twice for each biological cDNA samples after optimization, including negative and non-template controls.

### Data analysis and statistics

The threshold was manually determined as 0.025 in all reactions, and standards were calculated as follows: conc = 10ˆ (− 0.293*CT + 7.516); and CT values were calculated as follows: CT = − 3.410*log (conc) + 25.632, with an *R*^2^ value of 0.9963. The slope of the standard curve was determined to be − 3.410, and *R*2 = 0.99630. CT values were exported from the RT-PCR instrument after normalization via the ‘Dynamic Tube’ and ‘Slope Correction’ options of the RT-PCR software used. For determining fold change, samples were normalized using housekeeping genes *SNORD68, SNORD95* and *MIRTC* relevant for miRNA studies. Global mean normalization was used for Ct values and calculated concentrations were exported into the Excel spreadsheet, and the average value of duplicate Ct values was converted to quantities for analysis. The quality of expended mature miRNAs was checked via melt curve analyses using SYBR Green. Then, the Ct data were analyzed according to the fold-change (2(−ΔΔCT)) method and converted into fold regulation values using the online miScript miRNA PCR Array Data Analysis Tool (www.qiagen.com). *p* values under 0.05 were considered statistically significant.

## Results

Patient demographic data were shown in Table 1. In group 1, the mean age was 43.5 ± 14.13 (minimum age: 23, maximum age: 64). In group 2, the mean age was 60.3 ± 8.71 (minimum age: 50, maximum age: 77). In group 3, the mean age was 58 ± 7.89 (minimum age: 45, maximum age: 70). Gender for all 30 patients was male.

**Table 1:** Patient’s demographic data. (SD: Standard deviation). M: male

	Number of patient (n)	Gender	Age range	mean age ± SD
**Group 1**	10	male	23–64	43,5 ± 14,13
**Group 2**	10	male	50–77	60,3 ± 8,71
**Group 3**	10	male	45–70	58 ± 7,89

**Fig. 1:**
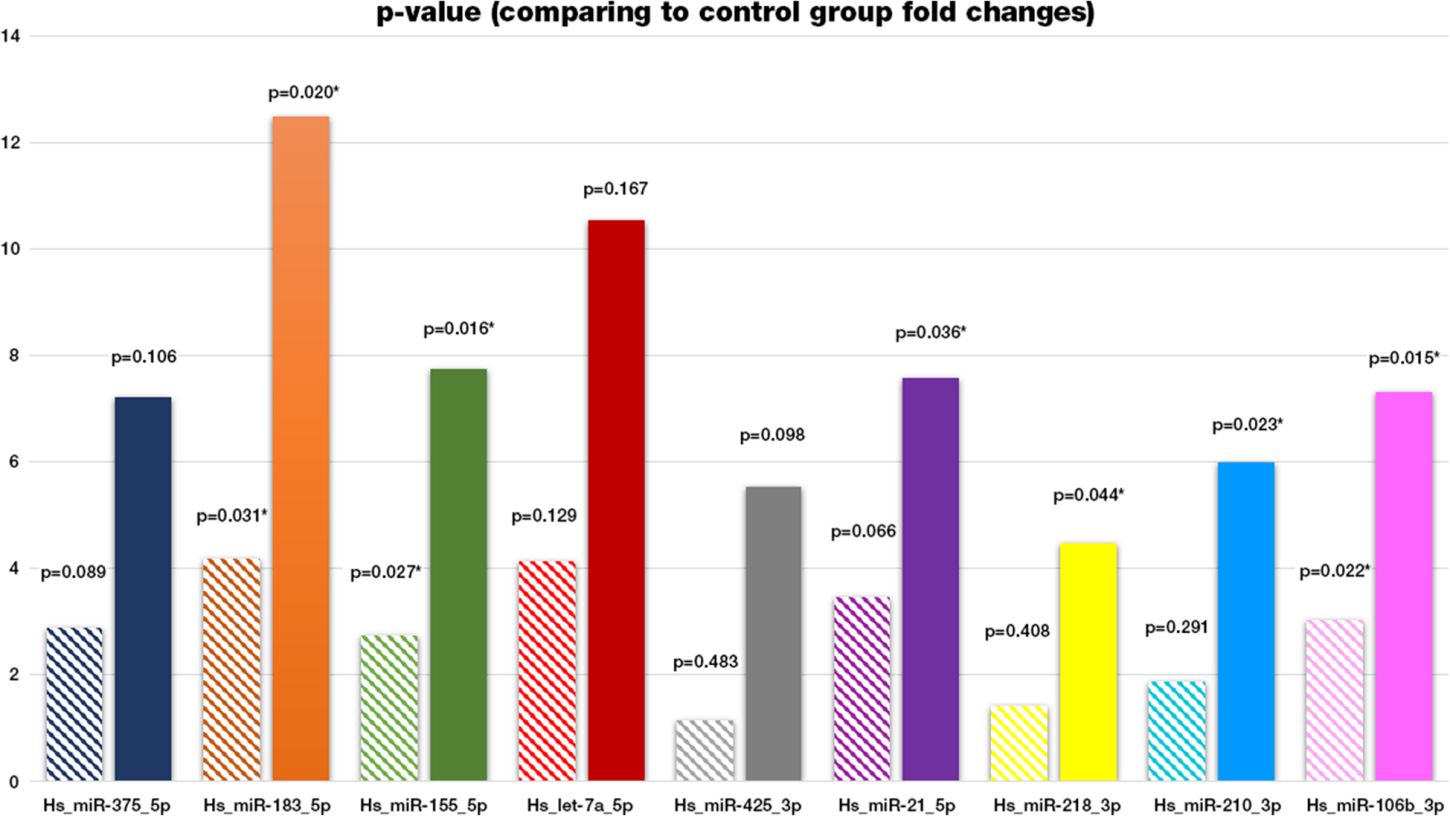
Clustered column chart of the premalignant and malignant group fold changes as compared to the benign group. Patterned columns shows the fold change values of the each miRNAs stated down below in the premalignant group. Solid filled columns represented the fold change values of the each stated miRNAs in malignant group

Fold regulations of the miRNA expressions in premalignant, malignant laryngeal lesions compared to benign lesions were given in Table 2 and Fig. 1.

**Table 2:** Fold regulations of the miRNA expressions in premalignant, malignant laryngeal lesions compared to benign lesions C.I. Confidence Interval

Genes	Fold Regulations (compared to benign group)					
	PREMALIGNANT GROUP			MALIGNANT GROUP		
	Fold Regulation	***p***-value	C.I. 95%	Fold Regulation	***p***-value	C.I. 95%
Hs_miR-375_5p	2.88	0.089235	(0.02, 5.74)	7,22	0.105618	(0.00001, 15.57)
**Hs_miR-183_5p**	**4.16**	**0.031872**	**(0.00, 8.32)**	**12.50**	**0.020141**	**(0.00001, 26.86)**
**Hs_miR-155_5p**	**2.72**	**0.027845**	**(0.55, 4.90)**	**7.75**	**0.016086**	**(0.00001, 15.73)**
Hs_let-7a_5p	4.12	0.129245	(0.39, 7.86)	10.55	0.167083	(0.00001, 22.11)
Hs_miR-425_3p	1.14	0.483030	(0.23, 2.05)	5.53	0.097976	(0.00001, 11.12)
Hs_miR-21_5p	3.45	0.066857	(0.01, 6.89)	**7.57**	**0.036338**	(0.00001, 16.88)
Hs_miR-218_3p	1.40	0.408852	(0.30, 2.50)	**4.45**	**0.044747**	(0.00001, 10.54)
Hs_miR-210_3p	1.86	0.291543	(0.00001, 4.03)	**5.98**	**0.023422**	(0.00001, 12.20)
**Hs_miR-106b_3p**	**3.01**	**0.021868**	(0.30, 5.73)	**7.31**	**0.015054**	(0.19, 14.44)

In the premalignant group *Hs_miR-375_5p, Hs_miR-183_5p, Hs_miR-155_5p, Hs_let-7a_5p, Hs_miR-21_5p* and *Hs_miR-106b_3p* expressions were upregulated; however, in comparison to benign group, only the overexpression of ***Hs_miR-183_5p, Hs_miR-155_5p*** **and** ***Hs_miR-106b_3p*** were statistically significant. (*p* < 0.05). The most upregulated miRNA was *Hs_miR-183_5p, whereas Hs_miR-155_5p* expression represented the lowest fold regulation (less than 2 folds).

***Hs_miR-375_5p, Hs_miR-183_5p, Hs_miR-155_5p, Hs_let-7a_5p, Hs_miR-425_3p, Hs_miR-21_5p, Hs_miR-218_3p, Hs_miR-210_3p*** **and** ***Hs_miR-106b_3p*** were all upregulated in the malignant group compared to benign group. However, in addition to significantly upregulated miRNAs in the premalignant group (*Hs_miR-183_5p, Hs_miR-155_5p, Hs_miR-106b_3p)*, overexpressions of ***Hs_miR-218_3p, Hs_miR-210_3p*** and ***Hs_miR-21_5p*** were also statistically significant. (*p* < 0.05). The most upregulated miRNA was *Hs_miR-183_5p,* whereas *Hs_miR-218_3p* was the lowest.

In other words, *Hs_miR-183_5p, Hs_miR-155_5p, and Hs_miR-106b_3p* expressions were statistically significant in both premalignant and malignant groups compared to the benign group, whereas *Hs_miR-21_5p, Hs_miR-218_3p, and Hs_miR-210_3p* expressions were statistically significant only in the malignant group.

When the malignant group was compared with the premalignant group, all miRNAs were upregulated in the malignant group. The most upregulated miRNA was *Hs_miR-425_3p whereas Hs_miR-106b_3p* was the lowest (Table 3).

**Table 3:** Overexpressed microRNAs in malignant laryngeal lesions in comparison with the premalignant laryngeal lesions

Gene Symbol	Fold Regulation
Hs_miR-375_5p	25,062
Hs_miR-183_5p	30,032
Hs_miR-155_5p	28,491
Hs_let-7a_5p	25,589
Hs_miR-425_3p	48,517
Hs_miR-21_5p	21,939
Hs_miR-218_3p	31,766
Hs_miR-210_3p	32,165
Hs_miR-106b_3p	24,259

**Fig. 2:**
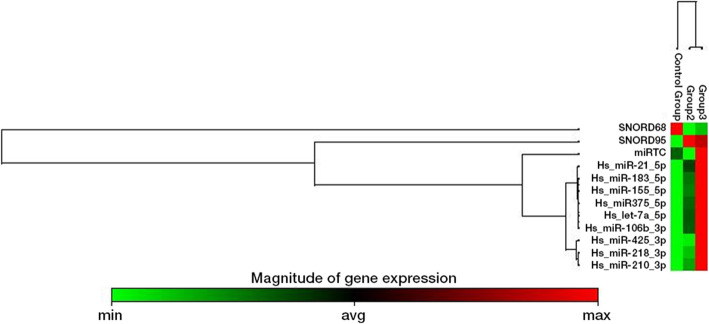
Clustergram of miRNA expressions between the groups. Control group (benign group), group 2 (Premalignant group), group 3 (malignant group) are the columns and miRNA expressions are showed for each group. *Green* represented standard gene expression whereas *red* represented overexpressed miRNAs

Overall, after normalization with *SNORD65* and *SNORD98* all miRNAs that we studied here represented upregulation as the laryngeal samples were transformed to malignant (Fig. 2). Among them, significant upregulations of *Hs_miR-183_5p, Hs_miR-155_5p* and *Hs_miR-106b_3p* were observed both in premalignant and malignant lesions whereas *Hs_miR-218_3p, Hs_miR-210_3p* and *Hs_miR-21_5p* were only seen in the malignant lesions compared to benign lesions.

## Discussion

The importance of miRNAs in gene regulation has emerged in recent years. After the first miRNA *Human_Lethal-7_5p (Hs_let-7a_5p)* was identified in *Caenorhabditis elegans*; to date, thousands of miRNAs have been discovered and many more remain unknown [10–12]. The numbers of miRNA studies conducted from an oncological perspective have increased steadily within the last decade and their role in malignant transformation is still a mystery [13–16]. Here, we investigated whether or not any miRNAs investigated here could be qualified as the transformation biomarkers within the laryngeal carcinoma progression.

*Hs_miR-106b_3p* is another miRNA that targets the 3’UTR of retinoblastoma *(RB)* gene [17] and upregulation of *RB* largely inhibited by *miR-106b* which further results in the induction of laryngeal carcinoma cells proliferation by deceased control of *RB* on G1/S transition of the cell cycle [17]. Though Xu et al. demonstrated a facilitated inhibition of cell proliferation and invasion due to the *Hs_miR-106b_3p* downregulation [18], Sun et al. identified a positive correlation between the upregulation of *Hs_miR-106b_3p* and lymph node metastasis, cancer stage in supraglottic laryngeal carcinoma [19]. In our study, compared to a benign group, we found 3.01 and 7.31 fold significantly increased *Hs_miR-106b_3p* expressions both in the premalignant and malignant groups, respectively (Table 2). However, when we compare the expressions in the malignant group with premalignant groups, *Hs_miR-106b_3p* expression was not statistically significant. Hence, our results could suggest that *Hs_miR-106b_3p* upregulation might be a transformation biomarker.

Similarly*, Hs_miR-155_5p* was also another miRNA that significant 2.72 and 7.75 fold increased expressions were noticed in the premalignant and malignant group compared to the benign group, respectively (Table 2). It has previously been studied in diverse malignancies, such as lung cancers, breast cancers and nasopharyngeal carcinomas [20, 21]. Among them, Shi et al. reported a positive correlation between *Hs_miR-155_5p* expression and poor prognosis in oral cavity malignancies [20]. Moreover, Zhao et al. reported higher levels of *Hs_miR-155_5p* expression in higher grade T3 and T4 laryngeal tumors than T2 laryngeal tumors [22]. The latter was also consistent with ours since about three times higher expressions were observed in the malignant group compared to the premalignant group. Hence, the malignancy potential could be attributable to *Hs_miR-155_5p* overexpression regardless of the other factors such as HPV related squamous cell carcinomas in the head and neck region [23]. In agreement with our study, results in the literature have primarily reported overexpression of *Hs_miR-155_5p*, nevertheless, Abigail et al. suggested that viral infections could change gene expressions and argued *that Hs_miR-155_5p* expression needed to be analyzed in larger series [23].

*Hs_miR-183_5p* on else has shown to play a role in cell differentiation, apoptosis, adhesion, and invasion. In colorectal, prostate and hepatic carcinomas, *Hs_miR-183_5p* was upregulated, whereas in ovarian and breast cancers including osteosarcomas it was downregulated [24]. There are few studies on laryngeal carcinomas for *Hs_miR-183_5p* expressions. Among them, Maia et al. compared radioresistant and radiosensitive laryngeal carcinomas but their results were insignificant [25]. In our study, compared to its expression in the benign group, *Hs_miR-183_5p* expression was increased by 4.16 and 12.50 fold in the premalignant and malignant group, respectively. Both groups generated statistically significant results (Table 2). However, *Hs_miR-183_5p* was known to target many enhancer transcription factors including histone deacetylase 2 (*HDAC2*). Downregulation of *the latter* has found to induce apoptosis and inhibit cellular proliferation and migration of laryngeal squamous carcinoma cells [26]. Moreover, a reverse correlation was reported with the metastatic potential of lung cancer cells. In contrast to these, miR-183 has a potential oncogenic role through the regulation of tumor suppressor genes such as *EGR1* and *PTEN* [27]. Hence, elevated expressions in the high-grade tumors might yield either as a compensatory mechanism to reduce the proliferation and migration through the repression of the transcription factors or to facilitate the tumor burden via the inhibition of the tumor suppressor genes. Whatever the reason why we have had a higher expression pattern in laryngeal carcinomas, the deregulation of this fundamental miRNA in its regulatory network may be significant imply on being the transformation biomarker for the laryngeal tumors.

Furthermore*, Hs_miR-21_5p* upregulations were supposed to be oncogenic for different kinds of tumors [28–36]. As a apoptosis regulating miRNA [37], *Hs_miR-21_5p* would be a prognostic marker in head and neck tumors that is relevant to our results in which the *Hs_miR-21_5p* was one of the three miRNAs that significantly upregulated solely in the malignant laryngeal tumors group (Table 2). Li et al. showed that in tongue squamous cell carcinoma, *Hs_miR-21_5p* was a prognostic marker, and by inhibiting *miR-21* with ASOs (antisense oligonucleotides), survival and growth of tumor cells were reduced, and apoptosis was induced [31]. On the other hand, Childs et al. found no correlation between any clinical parameters and *Hs_miR-21_5p* [32]. But, Avissar et al. reported a significant correlation between *Hs_miR-21_5p* expression and 5-year survival rates in the head and neck carcinomas [35]. Additionally, Lui et al. demonstrated that *Hs_miR-21_5p* was an oncomir which is overexpressed in laryngeal carcinomas compared to adjacent normal laryngeal tissue [38]. They reported a significant correlation between tumor stage, lymph node metastasis, tumor aggressiveness and survival [38]. In our study, compared to benign lesions, *Hs_miR-21_5p* expression was 3.45-fold and 7.57-fold increased in the premalignant and malignant group, respectively (Table 2). In the same way, Wei et al. obtained comparable results from a larger study representing *Hs_miR-21_5p* overexpression in premalignant and malignant laryngeal tissue samples in comparison with benign laryngeal tissues [39]. Though *Hs_miR-21_5p* overexpression was distinguishably significant only in the malignant group, hence, it might promote tumorigenesis by inhibiting apoptosis. We could not determine the exact reason but we have some potential explanations. First, we studied moderate-high-grade dysplasias as premalignant lesions, second due to pathological evaluation could be subjective and there was no certain consensus about grading of dysplasia. Third, Wei et al. has studied larger series than our that might have an impact.

The next miRNA which was significantly overexpressed only in the metastatic group was the *Hs_miR-218_3p* (Table 2). The latter was known to act as a tumor suppressor in several malignancies, such as renal cell carcinomas, lung cancers, and pancreatic carcinomas [40, 41]. Though limited studies were in the literature for laryngeal carcinomas, of the few that exist, Fukumoto et al. showed downregulation of *Hs_miR-218_3p* expression in laryngeal carcinomas. They identified that the silencing of *LOLX2 (lysyl oxidase-like 2)*, a target gene of *Hs_miR-218_3p*, inhibited migration and invasion in tumor cells [42]. Additionally, Takashi et al. reported it to regulate the migration and invasion of tumor cells via local adhesion pathways. They suggested that determining the mechanism of *Hs_miR-218_3p* could clarify the mechanism of local recurrence and distant metastasis [43]. Besides, Abigail et al. compared HPV-negative and HPV-positive head and neck cancers and showed significant downregulation of *miR-218* in the HPV-positive group [23]. In another study on cervical cancers, similar results were obtained for HPV-positive cervical cancers. In the same study, it was also shown that the downregulation of *Hs_miR-218_3p* by the *E6 oncogene* could cause overexpression of *LAMB3*, which is a target of *Hs_miR-218_3p* [23]. In opposite to these, Shi et al. highlighted the reversal of the growth inhibition in human gastric cancer cells caused by *miR-218* regulated *TFF1*. Likewise, overexpression of *Hs_miR-218_3p* in the malignant group might negatively regulate *TFF1* in an Erk1/2-dependent manner and promote malignancy as suggested [44]. However, we did not have the opportunity to analyse HPV status. In the literature, the majority of studies show downregulation of *Hs_miR-218_3p* in laryngeal carcinomas. Thus, we believe that unknown factors such as viral infections can affect the results, which should be investigated in larger series.

The discrepancies on *Hs_miR-210_3p* whether or not it is a tumor suppressor or an oncomir is still ongoing for many tumor types. Apart from that, Gee et al. indicated a correlation between the upregulation of *Hs_miR-210_3p* and poor prognosis in head and neck tumors by helping the vitality of tumor cells in hypoxic conditions [30]. Also it was associated with radiotherapy resistance in lung cancers in the same study [30]. In our study, compared to its expression in the benign group, *Hs_miR-210_3p* expression was significantly 7.31 fold increased in the malignant group (Table 2). However, only the overexpression of *Hs_miR-210_3p* in the malignant group was statistically significant (Table 2). Due to the challenging results in the literature more studies with larger series are needed to be investigated.

On the other hand, *Hs_miR-375_5p* has shown to be downregulated in malignancies including head and neck tumors [45, 46]. Overexpression of *Hs_miR-375_5p* reported to reduce cellular proliferation and migration in liver cells while stimulating both apoptosis and G1 arrest during the cell cycle [45]. Also, it was shown that *miR-375* overexpression could affect patient survival via reducing invasion by targeting *YAP1*, *JAK2* and phosphoinositide-dependent protein kinase-1(*PDK1)* [46]. Furthermore, Luo et al. enriched Harris’s suggestion and showed how *Hs_miR-375_5p* overexpression inhibited cell proliferation, migration, invasion and resulted in increased apoptosis via *IGF1R* expression [47]. Since the latter was a target of *miR-375*, increasing levels of *miR-375* expression could provide a significant reduction in *IGF1R* levels and its downstream signaling molecule *AKT* in laryngeal carcinoma cells [47]. Quaamari et al. also presented *PDK-1* as the target of *Hs_miR-375_5p* that contributed to *AKT* activation [48]. Despite all, Hu et al. identified a negative association between the alcohol use and *Hs_miR-375_5p* expression and indicated that the ratio of *miR-21/miR-375* had a 94% sensitivity and 94% specificity for distinguishing normal tissue from laryngeal carcinoma tissue [45].

Apart from that, Yu et al. discovered overexpression of *Hs_miR-106b_3p* and *Hs_miR-21_5p*, while downregulation of *Hs_miR-375_5p* expression in laryngeal carcinoma tissue compared to adjacent normal tissue [9]. Furthermore, the expression of *Hs_miR-106b_3p* and *Hs_miR-21_5p* expressions in poor and moderately differentiated laryngeal carcinomas were more upregulated than that in the benign and dysplastic laryngeal tissues [9]. Despite the results for *Hs_miR-106b_3p* and *Hs_miR-21_5p* were consistent with our data the downregulation of *Hs_miR-375_5p* expression particularly in advanced stages than in earlier stages was inconsistent [9]. Even though, its expression in the premalignant and malignant group were respectively 2.88 and 7.22 fold increased compared to the benign group, these values in our study were not statistically significant (Table 2).

In addition to this, *Hs_miR-425_3p* served as an oncomir and stimulated cell proliferation and inhibited apoptosis [49]. Furthermore, Li et al. found a correlation between *Hs_miR-425_3p* upregulation and lymph node metastasis in laryngeal carcinomas [50]. Notwithstanding that, in our study, *Hs_miR-425_3p* expressions were insignificantly increased (Table 2). In the same way, *Hs_let-7a_5p* expression was insignificantly 4.12-fold and 10.54-fold upregulated in premalignant and malignant laryngeal tissues when compared to benign laryngeal samples (Table 2). However, Long et al. has shown that *let-7a* expression was significantly downregulated in laryngeal squamous cell carcinomas compared to adjacent normal tissues and was significantly further decreased in non-differentiated carcinoma tissues compared with moderately and well-differentiated ones [13]. In contrast to them, in our study *let-7a* expression was insignificantly further upregulated as the tissues became more malignant. (Table 2, Fig. 2) Long et al. also identified 11 carcinoma samples (23%) demonstrating unchanged or even slightly elevated expression levels of *let-7a* compared with adjacent normal tissues [13]. They subsequently hypothesized that *let-7a* could have different impacts on different individuals or that let-7a may not take part in the pathogenesis of all laryngeal carcinomas [13]. In the light of this information, we believe that further investigations with larger series are needed particularly for those miRNAs who have insignificant results such as *Hs_miR-375_5p, Hs_miR-425_3p, and Hs_let-7a_5p.*

## Conclusion

Our study is one of the first to compare the expression levels of several different miRNAs between benign, premalignant and malignant laryngeal lesions with a relatively larger series upon the literature. They indicated that *Hs_miR-21_5p, Hs_miR-218_3p,* and *Hs_miR-210_3p* can be a potential biomarker for malignant laryngeal carcinomas. Furthermore, *Hs_miR-183_5p, Hs_miR-155_5p,* and *Hs_miR-106b_3p*, each upregulated both in premalignant and malignant groups compared to benign hyperplasia, might have a great value to help physicians to determine the malignancy potential of the laryngeal lesions as the transformation biomarkers upon prognosis.

## Data Availability

All data generated or analyzed during this study are included in this published article.
